# P-Selectin, Vascular Endothelial Cadherin, and Vascular Cell Adhesion Molecule-1 as Novel Biomarkers for ABO Hemolytic Disease of the Fetus and Newborn

**DOI:** 10.1155/ancp/9411137

**Published:** 2025-04-29

**Authors:** Weichun Tang, Linlin Zhu, Liwei Shi, Biao Gu

**Affiliations:** ^1^School of Medical Technology, Xinxiang Medical University, Xinxiang 453000, Henan, China; ^2^The Third People's Hospital of Bengbu Affiliated to Bengbu Medical University, Bengbu 233030, Anhui, China

**Keywords:** ABO-HDFN, PS, VCAM-1, VE-Cad

## Abstract

**Objective:** This study aims to assess the potential of vascular endothelial injury markers, namely, P-selectin (PS), vascular endothelial cadherin (VE-Cad), and vascular cell adhesion molecule-1 (VCAM-1), as diagnostic and prognostic biomarkers for ABO hemolytic disease of the fetus and newborn (HDFN).

**Methods:** A total of 218 pregnant women with ABO blood group incompatibility were recruited from the Third People's Hospital of Bengbu Affiliated to Bengbu Medical University. The serum levels of PS, VCAM-1, and VE-Cad were measured, and the participants were followed up until postpartum. The women were divided into an HDFN group and a control group based on the occurrence of ABO-HDFN. The correlations between the three vascular endothelial injury markers, pregnant anti-A/B antibody titers, and the occurrence and severity of HDFN were analyzed.

**Results:** Compared to the control group, the levels of PS, VCAM-1, and VE-Cad were significantly elevated in the HDFN group. Additionally, these markers increased with higher IgG anti-A/B titers. For diagnosing HDFN, the area under the curve (AUC) for PS, VCAM-1, and VE-Cad were 0.826, 0.765, and 0.799, respectively. Moreover, the combined AUC of the three markers with IgG anti-A/B titers was 0.9. The levels of the three biomarkers were significantly negatively correlated with neonatal hemoglobin (Hb) and significantly positively correlated with reticulocyte percentage (Ret%), indirect bilirubin (IBIL), and lactate dehydrogenase (LDH). Univariate logistic regression indicated that increased levels of PS, VCAM-1, and VE-Cad were associated with a higher probability of ABO-HDFN. Multivariate logistic regression revealed that PS is an independent positive factor for HDFN.

**Conclusion:** PS, VCAM-1, and VE-Cad provide experimental evidence for prenatal screening, diagnosis, early prevention and treatment of ABO-HDFN.

## 1. Introduction

Hemolytic disease of the fetus and newborn (HDFN) primarily refers to an immune-mediated hemolytic condition caused by blood type incompatibility between the mother and fetus, where pregnant IgG antibodies sensitize fetal or neonatal red blood cells, leading to hemolysis [1]. Clinically, ABO-HDFN presents with severe anemia, hypoproteinemia, and heart failure, which can result in fetal hydrops or even stillbirth. Within hours or days following birth, newborns or premature infants may develop varying degrees of jaundice, anemia, and hepatosplenomegaly [2]. The onset of ABO-HDFN is unpredictable, which occurs during either the fetal or neonatal period and exhibits a wide array of clinical symptoms, rendering early detection challenging. Consequently, early diagnosis and effective prognostic evaluation are paramount for the prevention and treatment of ABO-HDFN.

In recent years, vascular endothelial injury markers have garnered significant attention for their role in the diagnosis and prognostic evaluation of a variety of diseases. Research has reported that CD144^+^ microparticles can function as indicators of vascular endothelial injury in neonates experiencing ABO blood type incompatibility hemolysis, showing a positive correlation with the severity of the condition [3]. Additional studies have suggested that increased levels of endothelial microparticles in children with ABO-HDFN may serve as surrogate markers for vascular dysfunction and the severity of the disease [4]. This implies that pregnant anti-A and anti-B antibodies could potentially target not only the corresponding antigens on red blood cells to provoke hemolysis but also the A and B blood group antigens on the surface of vascular endothelial cells, leading to endothelial damage. Vascular endothelial cells are crucial for sustaining vascular permeability, modulating blood flow, ensuring anticoagulation, and managing inflammatory responses. Upon injury to these cells, they release a range of markers, such as P-selectin (PS), vascular endothelial cadherin (VE-Cad), and vascular cell adhesion molecule-1 (VCAM-1). These markers not only reflect the extent of endothelial cell damage but are also closely linked to the onset and progression of various vascular-related diseases [5–7].

This research amalgamates the surveillance outcomes of pregnant IgG antibody titers and monitors the alterations in serum concentrations of PS, VCAM-1, and VE-Cad, examining their association with the emergence and progression of ABO-HDFN. The findings offer empirical support for the prenatal diagnosis and early intervention strategies of ABO-HDFN.

## 2. Methods and Materials

### 2.1. Case Collection

This study was approved by the Institutional Ethics Committee of the Third People's Hospital of Bengbu Affiliated to Bengbu Medical University. All participants provided written informed consent. The cases of pregnant women were compiled from the Third People's Hospital of Bengbu Affiliated to Bengbu Medical University, with study subjects being chosen in accordance with specific inclusion and exclusion criteria. The inclusion criteria are as follows: (1) pregnant women with blood type O and RhD positive, whose partners possess a non-O blood type and are RhD positive, and whose newborns exhibit a non-O blood type, indicating ABO blood type incompatibility between mother and child. (2) Newborns must be singletons. (3) All participants must have a comprehensive understanding of the study and provide signed informed consent prior to enrollment. The exclusion criteria are as follows: (1) newborns diagnosed with hemolytic diseases attributed to blood group systems other than ABO; (2) infants with hemolytic diseases resulting from non-blood group–related causes; (3) pregnant women with underlying diseases affecting the heart, liver, kidneys, or brain; and (4) pregnant women with positive results for irregular antibody screening. Utilizing these criteria and subsequent data, the study ultimately enrolled 218 pregnant women and their respective newborns. The pregnant plasma samples used in this study were all collected during late pregnancy (upon hospital admission for delivery).

### 2.2. Instruments and Reagents

They were as follows: XN-10 Hematology Analyzer (Sysmex Corporation, Japan) and AU5800 Automatic Biochemical Analyzer (Beckman Coulter, Inc.); centrifuge system, cassette incubator (Bio-Rad, USA), irregular antibody screening cells, human ABO red blood cells, neonatal hemolytic disease detection cards, and ABO and Rh monoclonal antibody blood type detection cards (Changchun Boxun Biotechnology Co., Ltd.); IgG anti-A/B titer detection kits (Shanghai Blood Biological Medicine Co., Ltd.); and PS, VE-Cad, and VCAM-1 assay kits (Shanghai Yanzun Biotechnology Co., Ltd.).

### 2.3. Blood Type Identification and Irregular Antibody Screening

Anticoagulated venous whole blood samples were collected from pregnant women and newborns and subsequently centrifuged at 1000 g for 3 min to segregate the red blood cells from the plasma for further analysis. The presence of irregular antibodies, as well as the Rh and ABO blood types, was determined using microcolumn gel card techniques.

### 2.4. Hemolysis Tests and Hemolysis Indicator Collection

The collected samples of anticoagulated venous whole blood from newborns were washed and subsequently subjected to three hemolysis tests: the direct antiglobulin test (DAT), the free antibody test, and the elution test. A positive DAT was indicated by agglutination, whereas the free antibody and elution tests were deemed positive upon the detection of antibodies reacting with neonatal red blood cells. A positive result in the elution test served to confirm the diagnosis of ABO-HDFN. Additional hemolysis indicators monitored in newborns comprised hemoglobin (Hb), reticulocyte percentage (Ret%), indirect bilirubin (IBIL), and lactate dehydrogenase (LDH) levels.

### 2.5. IgG Anti-A/B Titer Determination

To ascertain IgG anti-A/B titers, 100 μL of pregnant plasma was combined with an equal volume of β-mercaptoethanol, followed by incubation at 37°C for 15 min. Subsequently, the mixture was serially diluted using physiological saline. ABO reagent red blood cells, matching the father's blood type, were introduced to the mixture, which was then incubated at 37°C for an additional 30 min. Following this, the IgG blood group antibody titers were quantified. The experimental outcomes were computed by plotting the optical density (OD) values of the standards on the *x*-axis against the concentration values on the *y*-axis to generate a standard curve. The linear regression equation obtained from this curve was subsequently employed to determine the sample concentrations based on their respective OD values.

### 2.6. Detection of PS, VCAM-1, and VE-Cad

Five milliliters of venous whole blood were collected from pregnant women using serum separation tubes and permitted to stand at room temperature for a duration of 2 h. Subsequently, the samples underwent centrifugation at a force of 1000×*g* for a period of 20 min, after which the supernatant was carefully extracted and either stored at −20°C or −80°C. Enzyme-linked immunosorbent assays (ELISAs) were conducted in accordance with the manufacturer's guidelines to quantify the levels of PS, VCAM-1, and VE-Cad.

### 2.7. Statistical Analysis

The statistical analyses and visualizations presented in this study were executed using R software, version 4.3.0. The specific R packages and their respective versions utilized were ggplot2 (3.3.6), stats (4.2.1), car (3.1-0]) ggbeeswarm (0.7.2), and pROC (1.18.0). Student's *t*-test was applied to compare continuous variables between two groups, whereas the chi-square test or Kruskal–Wallis test was used for comparisons across multiple groups. Spearman's test was conducted to analyze the correlation between two groups. Univariate and multivariate logistic regression analyses were employed to pinpoint risk factors associated with ABO-HDFN. Receiver operating characteristic (ROC) curves were utilized to ascertain the cutoff values and the area under the curve (AUC) for PS, VCAM-1, and VE-Cad in diagnosing HDFN. A *p*-value of less than 0.05 was deemed statistically significant, with the following notation: *⁣*^*∗*^*p* < 0.05, *⁣*^*∗∗*^*p* < 0.01, and *⁣*^*∗∗∗*^*p* < 0.001.

## 3. Results

### 3.1. PS, VCAM-1, and VE-Cad Are Significantly Elevated in Pregnant Women With HDFN

**Figure 1:**
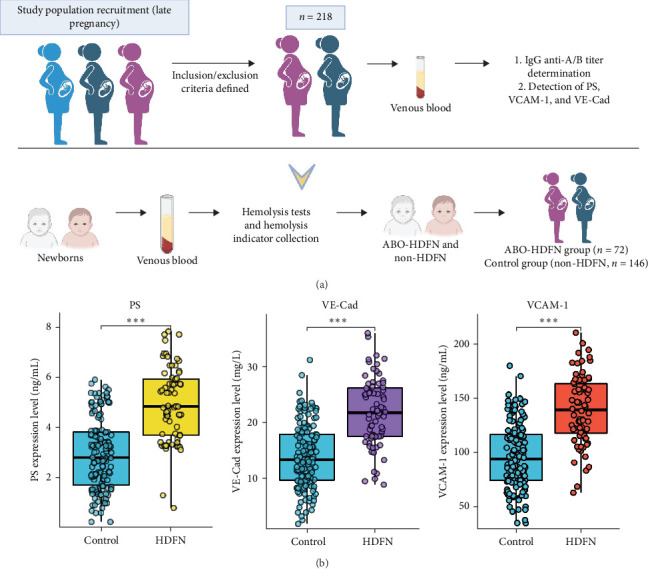
(A) This study includes a flowchart outlining the sample processing procedures and experimental group allocations. (B) Analysis of the differences in PS, VE-Cad, and VCAM-1 between the control group and the HDFN group (*⁣*^*∗*^*p* < 0.05, *⁣*^*∗∗*^*p* < 0.01, and *⁣*^*∗∗∗*^*p* < 0.001).

Based on the defined inclusion and exclusion criteria, this study enrolled a total of 218 pregnant women in late pregnancy. According to the postpartum neonatal hemolysis tests, with the positive elution test serving as the definitive diagnostic criterion for ABO-HDFN, the subjects were divided into two groups: the ABO-HDFN group (*n* = 72) and the control group (non-HDFN, *n* = 146). The maternal blood samples were collected during late pregnancy. Details of sample collection, processing, and group allocation are illustrated in Figure 1A. A comprehensive analysis was conducted on various clinical indicators pertaining to both the expectant mothers and their newborns. Based on their median values, endothelial injury markers, specifically PS, VCAM-1, and VE-Cad, were classified into two distinct categories: high-expression (high) and low-expression (low). The chi-square test revealed no significant disparities in age, parity, delivery method, gestational age (in weeks), or newborn gender between the HDFN and control groups. Nevertheless, notable correlations were observed between IgG anti-A/B titers and endothelial injury markers (PS, VCAM-1, and VE-Cad) as detailed in Table 1. In comparison to the control group, the ABO-HDFN group exhibited significantly higher levels of endothelial injury markers (PS, VCAM-1, and VE-Cad) as detailed in Figure 1B.

### 3.2. PS, VCAM-1, and VE-Cad Increase With Rising IgG Anti-A/B Titers

**Figure 2:**
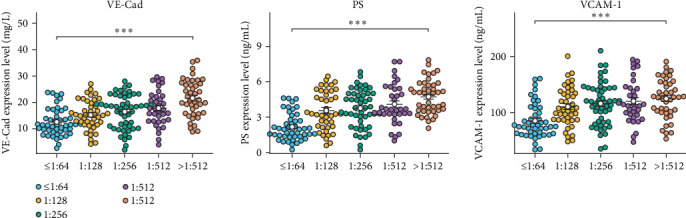
Analysis of differences in PS, VCAM-1, and VE-Cad across five IgG anti-A/B titer groups (*⁣*^*∗*^*p* < 0.05, *⁣*^*∗∗*^*p* < 0.01, and *⁣*^*∗∗∗*^*p* < 0.001).

To examine the correlation between PS, VCAM-1, VE-Cad, and IgG anti-A/B titers, the IgG anti-A/B titers were stratified into five categories: ≤1:64 (*n* = 46), 1:128 (*n* = 43), 1:256 (*n* = 52), 1:512 (*n* = 35), and >1:512 (*n* = 42). Our observations revealed that the concentrations of PS, VCAM-1, and VE-Cad exhibited a significant increase corresponding with the escalation of IgG anti-A/B titers (Figure 2).

### 3.3. PS, VCAM-1, and VE-Cad as Potential Diagnostic Markers for ABO-HDFN

**Figure 3:**
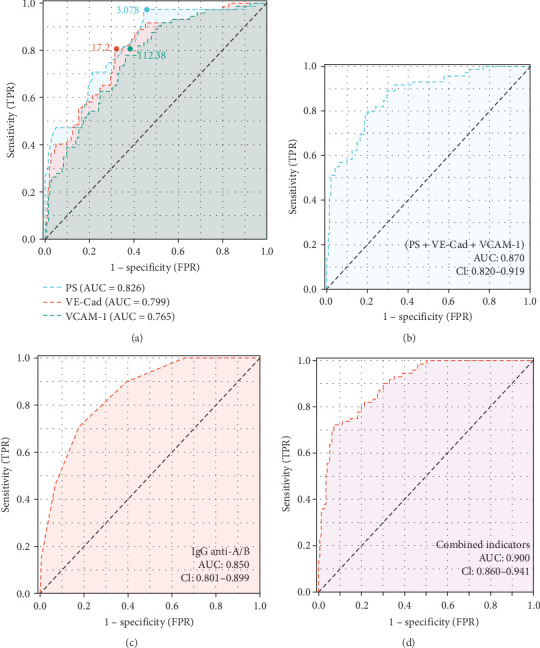
PS, VCAM-1, and VE-Cad as potential diagnostic markers for ABO-HDFN. (A) ROC curves and cutoff values for PS, VCAM-1, and VE-Cad. (B) ROC curve analysis of the combined diagnostic value of PS, VE-Cad, and VCAM-1 for ABO-HDFN. (C) ROC curve for IgG anti-A/B titer. (D) Combined ROC curve for PS, VCAM-1, VE-Cad, and IgG anti-A/B titer.

To assess the diagnostic efficacy of PS, VCAM-1, and VE-Cad in ABO-HDFN, a ROC analysis was conducted. The AUC values for PS, VCAM-1, and VE-Cad were determined to be 0.826, 0.765, and 0.799, respectively (Figure 3A). The optimal cutoff value for PS was established at 3.087 ng/mL, yielding a specificity of 0.719 and a sensitivity of 0.736. In the case of VCAM-1, the cutoff value was set at 112.38 ng/mL, achieving a specificity of 0.616 and a sensitivity of 0.806. For VE-Cad, the cutoff value was determined to be 17.2 mg/L, with a specificity of 0.819 and a sensitivity of 0.678. The combined diagnostic AUC of these three biomarkers was 0.870 (Figure 3B). Furthermore, the AUC for IgG anti-A/B titer was found to be 0.850 (Figure 3C), and the aggregate diagnostic AUC for PS, VCAM-1, VE-Cad, and IgG anti-A/B titer was calculated to be 0.900 (Figure 3D).

### 3.4. Increased Levels of PS, VCAM-1, and VE-Cad Are Associated With a Higher Probability of ABO-HDFN

**Figure 4:**
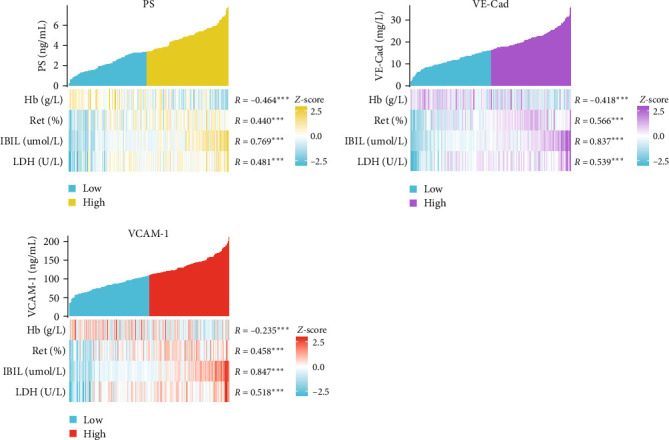
Correlation analysis of PS, VCAM-1, and VE-Cad levels with neonatal hemolysis indicators (Hb, Ret%, IBIL, and LDH).

We examined the correlation between the expression levels of PS, VCAM-1, VE-Cad, and neonatal hemolysis indicators, including Hb, Ret%, IBIL, and LDH. Our findings indicated that the expression levels of PS, VCAM-1, and VE-Cad exhibited a significant negative correlation with neonatal Hb and a significant positive correlation with Ret%, IBIL, and LDH (Figure 4). Moreover, this study further analyzes whether there are significant differences in the probabilities of ABO-HDFN occurrence based on 12 clinical indicators. Among these indicators, neonatal hemolysis parameters (Hb, Ret%, IBIL, and LDH), PS, VCAM-1, and VE-Cad are categorized into high-expression and low-expression groups based on their median expression levels. In this study, subsequent univariate logistic regression analysis revealed that pregnant IgG anti-A/B titer (1:512 group and >1:512 group) and three endothelial injury markers (PS, VCAM-1, and VE-Cad) were significantly associated with the incidence of ABO-HDFN (*p*  < 0.05). Specifically, the odds ratio (OR) values for these variables were all greater than 1, indicating a positive correlation with the occurrence of ABO-HDFN (Table 2). Further multivariate logistic regression analysis demonstrated that pregnant IgG anti-A/B titer (>1:512 group) and PS were independent predictors of ABO-HDFN (Table 2). This indicates that, after adjusting for other variables, increased levels of pregnant IgG anti-A/B titer >1:512 and PS significantly elevate the risk of ABO-HDFN. In conclusion, both univariate and multivariate logistic regression analyses indicate that elevated levels of PS, VCAM-1, and VE-Cad are associated with an increased probability of ABO-HDFN, with PS being an independent positive influencing factor.

## 4. Discussion

ABO-HDFN is an immune-mediated hemolytic disorder caused by ABO blood group incompatibility between the mother and the fetus or neonate. Clinically, it can manifest as fetal hydrops, early miscarriage, or even stillbirth, while neonates or preterm infants may present with varying degrees of jaundice, anemia, or hepatosplenomegaly. In cases of rapid disease progression without distinctive clinical signs, infants are at high risk of developing hyperbilirubinemia, which can lead to bilirubin encephalopathy in severe situations and pose a life-threatening danger [8–10]. Consequently, severe presentations of ABO-HDFN are frequently reported. Studies have shown that early screening, early diagnosis, and early intervention are pivotal to improving the prognosis of ABO-HDFN [11, 12].

Currently, the diagnosis of ABO-HDFN relies on collecting neonatal blood samples for three hemolysis tests: the DAT, the free antibody test, and the red blood cell antibody elution test. Additionally, Hb and bilirubin levels are also measured [1, 13]. This procedure is time-consuming, typically requiring 2–3 days to obtain results, and the hemolysis tests can yield false positives and false negatives, which hinders the early diagnosis of ABO-HDFN [14]. Prenatal screening for ABO-HDFN primarily involves conducting ABO blood typing and measuring IgG anti-A/B antibody titer. The pregnant serum IgG anti-A/B titer is a significant predictor for assessing the risk of HDFN in the newborn [15]. The tube method, although it is the most classical and reliable technique for detecting pregnant serum IgG anti-A/B titers, is also complex and time-consuming. It is challenging to automate and presents difficulties in accurately interpreting the results [16]. Krog et al. enhanced the prediction of ABO-HDFN via flow cytometric characterization of maternal anti-A and anti-B antibodies [17]. Meanwhile, Robert et al. employed ETCOc measurements for the diagnosis and management of various perinatal/neonatal hemolytic disorders [18]. Thus far, no systematic screening protocol for ABO-HDFN has been established in clinical practice, nor have there been sufficiently precise preventive measures. Consequently, ABO-HDFN continues to pose a major threat to the health and survival of fetuses and neonates [12, 19, 20]. Prevention continues to be crucial in reducing the incidence and mortality associated with ABO-HDFN, underscoring the importance of identifying precise biomarkers for prenatal diagnosis.

Endothelial cells are crucial for sustaining vascular permeability, modulating blood circulation, and mediating inflammatory responses. Researches indicate that the vascular endothelium sustains injury in infants afflicted with ABO-HDN, and the extent of damage is positively correlated with the severity of the condition [3, 4]. ABO immune complexes, formed by the interaction of ABO-incompatible antigens and antibodies, can compromise the integrity of the endothelial cell barrier [21]. When endothelial cells sustain damage, they secrete indicators including PS, VE-Cad, and VCAM-1 [22–24]. Previous studies have primarily focused on endothelial microparticles (such as CD144+ particles) as surrogate markers of endothelial damage in neonatal ABO-HDFN [3, 4]. However, soluble biomarkers reflecting direct endothelial activation remain relatively underexplored in this context. This study assessed the diagnostic and prognostic significance of endothelial injury markers, including PS, VE-Cad, and VCAM-1, in the ABO-HDFN. Our investigation revealed that the levels of these markers were markedly increased in the HDFN group, indicating that endothelial cell injury might be a critical factor in the development of HDFN. PS, a selectin family member, is predominantly expressed on the surface of endothelial cells and platelet granules, serving as a pivotal molecule for recognizing activated platelets and facilitating thrombosis [25]. Typically, PS is expressed at low levels. However, its concentration in the bloodstream increases sharply when platelets and endothelial cells are activated by inflammatory mediators. As a result, PS acts as a marker for endothelial cell injury [26]. Surface-exposed PS can initiate interactions between leukocytes and either endothelial cells or platelets, thereby directing leukocytes toward sites of inflammation [27]. In addition, PS plays a pivotal role in thrombosis; its elevated expression is closely linked to platelet activation and serves as a marker of prothrombotic status [28]. VE-Cad is a molecule that is uniquely expressed in vascular endothelial cells, playing a crucial role in facilitating cell–cell contact and adhesion. It is essential for the development of the vascular system and the regulation of vascular permeability [29, 30]. VE-Cad forms homodimers with adjacent VE-Cad molecules via its extracellular domain, thereby establishing stable intercellular junctions that constitute the foundation of the vascular endothelial barrier [31]. Damage to the endothelium disrupts its adhesive function, resulting in the release of soluble VE-Cad, capillary leakage, tissue edema, and organ dysfunction [29]. VCAM-1, a vital component of the adhesion molecule superfamily, is typically expressed at low levels under normal circumstances [32]. However, it assumes a pivotal role in immune and inflammatory processes. It facilitates the adhesion of leukocytes to endothelial cells and acts as an indicator of endothelial damage. Consequently, serum levels of VCAM-1 markedly increase in response to vascular injury [33]. The marked increase in these indicators suggests that HDFN could trigger a cascade of pathophysiological alterations, potentially via the activation and damage of endothelial cells.

Our study is the first to identify and emphasize that vascular endothelial injury markers—PS, VE-Cad, and VCAM-1—are novel biomarkers specifically associated with ABO-HDFN. This study further analyzed the correlation between three markers of endothelial injury and pregnant anti-A/B antibody titers, the incidence of HDFN, and its severity. The findings indicated that as the titers of IgG anti-A/B antibodies rose, so did the expression levels of the three markers. Elevated pregnant serum IgG anti-A/B titers in pregnancies with O blood type were linked to a higher incidence of ABO-HDFN [14]. The findings indicated that pregnant anti-A/B antibodies could play a role in the development of HDFN by inducing damage to endothelial cells. Furthermore, the study evaluated the diagnostic efficacy of three markers of endothelial injury in HDFN, determining that the areas under the ROC curves (AUCs) for PS, VCAM-1, and VE-Cad were 0.826, 0.765, and 0.799, respectively, suggesting a high degree of diagnostic precision. The integration of these markers alongside IgG anti-A/B titers resulted in an AUC of 0.9, indicating an even greater diagnostic value. This implies that the employment of multiple markers can improve the accuracy of HDFN diagnosis, offering a novel approach for early clinical screening and diagnosis. This practical application could streamline early clinical screening, facilitating more accurate risk stratification and timely intervention. Additionally, Hb, Ret, IBIL, and LDH are indicators of ABO-HDFN severity [34]. The three markers were significantly negatively correlated with neonatal Hb and positively correlated with Ret, IBIL, and LDH. These findings further underscored the significance of endothelial damage in HDFN and indicated that these biomarkers might function as prospective indicators for assessing the severity of HDFN. In prognostic evaluation, univariate logistic regression analysis showed that PS, VCAM-1, and VE-Cad were adverse prognostic factors for HDFN, while multivariate logistic regression analysis indicated that PS was an independent adverse prognostic factor. This highlights the significant independent prognostic value of PS in HDFN, offering crucial prognostic insights for clinical practice. To the best of our knowledge, this is the first study to investigate these specific endothelial injury markers in the context of ABO-HDFN, highlighting their novelty and potential clinical utility. They demonstrated strong diagnostic value both individually and when combined with antibody titers, suggesting their promise for prenatal prediction, early diagnosis, and risk stratification of ABO-HDFN.

Despite demonstrating the pivotal role of endothelial injury markers in the diagnosis and prognosis of ABO-HDFN, this study has several limitations. First, the relatively small sample size constrains the statistical power, making it necessary to conduct larger, multicenter trials to validate and generalize our findings. Second, as the study was conducted at a single institution, the results may not be representative of broader patient populations with diverse clinical backgrounds. Third, owing to its cross-sectional design, this investigation did not capture the temporal changes of these biomarkers throughout gestation or postpartum. Consequently, more robust time-to-event analyses (e.g., Kaplan–Meier or Cox regression) could not be performed. Future longitudinal research is needed to delineate the causal relationship between endothelial injury markers and ABO-HDFN progression, as well as to refine their predictive utility in different clinical settings.

## 5. Conclusion

In summary, this study illustrates that endothelial injury markers such as PS, VE-Cad, and VCAM-1 are of considerable significance for the diagnosis and prognostic evaluation of ABO-HDFN. Employing these markers in conjunction can enhance diagnostic precision and furnish vital prognostic data for clinical practice. These findings provide experimental support for the prenatal screening, diagnosis, and early intervention of ABO-HDFN, carrying significant clinical ramifications. Future research should further validate the application of these markers across diverse populations and delve into their potential mechanisms for the intervention and treatment of HDFN.

## Figures and Tables

**Table 1:** Comparison of clinical data of pregnant women and neonates.

Research projects	HDFN group	Control group	*χ*2	*p*-Value
Age	—	—	3.397	0.065
18–29	26	72	—	—
30–40	46	74	—	—
Number of pregnancies	—	—	2.77	0.096
1 time	25	68	—	—
≥2 times	47	78	—	—
Mode of delivery	—	—	0.086	0.769
Cesarean section	33	70	—	—
Vaginal delivery	39	76	—	—
Gestational age (weeks)	—	—	0.134	0.714
≥37	68	136	—	—
<37	4	10	—	—
Sex of newborn	—	—	—	—
Male	39	70	0.747	0.387
Female	33	76	—	—
IgG anti-A/B titer	—	—	14.363	0.006
≤1:64	6	40	—	—
1:128	14	29	—	—
1:256	20	32	—	—
1:512	18	17	—	—
>1:512	14	28	—	—
PS (cutoff values: 3.400 ng/mL)	—	—	40.15	<0.0001
High	58	51	—	—
Low	14	95	—	—
VE-Cad (cut-off values: 16.191 ng/mL)	—	—	16.259	0.0001
High	50	59	—	—
Low	22	87	—	—
VCAM-1 (cut-off values: 108.398 ng/mL)	—	—	14.019	0.0002
High	49	60	—	—
Low	23	86	—	—

**Table 2:** Univariate/multivariate logistic regression analysis of clinical parameters of pregnant women and neonates.

Characteristics	Total (*N*)	Univariate analysis		Multivariate analysis	
		Odds ratio (95% CI)	*p* Value	Odds ratio (95% CI)	*p* Value
Age	218	—	—	—	—
18–29	98	Reference	—	Reference	—
30–40	120	1.721 (0.964–3.075)	0.066	0.963 (0.461–2.013)	0.921
Mode of delivery	218	—	—	—	—
Cesarean section	103	Reference	—	Reference	—
Natural childbirth	115	1.089 (0.618–1.917)	0.769	1.101 (0.452–2.678)	0.832
Gestational age (weeks)	218	—	—	—	—
≥37	204	Reference	—	Reference	—
<37	14	0.772 (0.331–1.803)	0.551	1.299 (0.417–4.052)	0.652
Sex of newborn	218	—	—	—	—
Male	109	Reference	—	Reference	—
Female	109	1.181 (0.671–2.078)	0.565	1.069 (0.850–1.344)	0.569
IgG anti-A/B titer	218	—	—	—	—
≤64	46	Reference	—	Reference	—
1:128	43	2.138 (0.799–5.719)	0.130	1.260 (0.812–1.955)	0.301
1:256	52	2.364 (0.670–8.339)	0.181	1.373 (0.392–4.804)	0.620
1:512	35	4.167 (1.497–11.600)	0.006	1.850 (0.554–6.178)	0.317
>1:512	42	7.059 (2.387–20.875)	<0.001	5.010 (1.399–17.934)	0.013
Hb	218	—	—	—	—
High	109	Reference	—	Reference	—
Low	109	1.517 (0.859–2.679)	0.151	1.048 (0.696–1.579)	0.820
Ret	218	—	—	—	—
Low	109	Reference	—	Reference	—
High	109	1.181 (0.671–2.078)	0.565	1.014 (0.998–1.031)	0.088
IBIL	218	—	—	—	—
Low	109	Reference	—	Reference	—
High	109	1.283 (0.729–2.260)	0.388	0.808 (0.519–2.560)	0.344
LDH	218	—	—	—	—
Low	109	Reference	—	Reference	—
High	109	1.283 (0.729–2.260)	0.388	1.281 (0.521–3.169)	0.591
PS	218	—	—	—	—
Low	109	Reference	—	Reference	—
High	109	2.559 (1.112–5.887)	0.002	1.287 (1.026–1.614)	0.029
VE-Cad	218	—	—	—	—
Low	109	Reference	—	Reference	—
High	109	1.954 (1.256–3.041)	0.027	1.097 (0.716–1.681)	0.665
VCAM-1	218	—	—	—	—
Low	109	Reference	—	Reference	—
High	109	1.584 (1.069–2.347)	0.021	0.669 (0.098–4.566)	0.682

## Data Availability

The data that support the findings of this study are available on request from the corresponding author. The data are not publicly available due to privacy or ethical restrictions.
